# A Calling to Professionalism

**DOI:** 10.15694/mep.2017.000218

**Published:** 2017-12-06

**Authors:** Patrick Herron

**Affiliations:** 1 Albert Einstein College of Medicine

**Keywords:** Bioethics, Professionalism, Coaching, Medical Ethics

## Abstract

This article was migrated. The article was marked as recommended.

The editor for this theme issue on Accessing Medical Education has asked authors to reflect on how they came to be the medical education professional they are today. My own pathway was non-traditional to say the least. Some professions are sought out and others come about through serendipitous moments and opportunities. My own journey has contributed to my current scholarly focus on professionalism in health care.

## A Calling to Professionalism

I am a Bioethicist and I won’t be offended or surprised if you have the urge to Google that term before reading further. There are many definitions. When I am asked in the course of casual conversation about the work I do, I reply that I teach Bioethics. Frequently this is met with slow head nods of uncertainty and so clarify further that I specialize in teaching about ethical issues concerning health care practice, policy and the law. This is by no means comprehensive of what a bioethicist does or what I do in the course of my own work, but as an introvert it serves me well when meeting new people for the first time. Despite my socially inward tendencies, teaching is what I love to do and has always been the type of work to give me the greatest enjoyment. Recently I was asked to reflect on my teaching philosophy and what emerged after weeks of procrastination was a description similar to that of being a coach. After putting words to paper, I began to see my perspective of teaching had always been present in my work and approach to learning although I had not identified it as coaching.

Bioethics was not my first profession. It was one that I serendipitously found myself drawn to many years ago. After several years of working in public education as a special education teacher, I accepted what I had thought would be a temporary position in medical education. Through my work supporting physician educators with teaching and curricular development, I became aware of the emerging field of bioethics. I found the application of philosophy and exploration of values in regards to medicine to be fascinating and a field of study that was both exciting and challenging. In the past three decades, education in ethics has become an integral part of medical education and has received particular attention in recent years because of the increasing emphasis placed on professional formation by accrediting bodies such as the Liaison Committee on Medical Education and the Accreditation Council for Graduate Medical Education ( Carrese et al 2015).

It would take many years of balancing part-time academic study with full-time professional duties in order to complete my doctorate in clinical bioethics. My interest and professional goal in pursuing advanced study was to further my ability to practice bioethics in clinical settings and enhance my teaching skills. The opportunity to not only educate, but inspire others to appreciate the value and importance of ethics analysis in their clinical work is a wonderfully satisfying feeling. My love of teaching is not limited to bioethics though. I have taught in and served as a course leader in a doctoring course for medical students for the past seven years. Helping novice students to develop the confidence and clinical skills required to engage with patients and colleagues has been equally rewarding to me professionally and personally. In the course of this work, questions related to bioethics frequently arise. I cherish those teachable moments when I can call upon my own knowledge and experience to further engage with my students. My work has also included examining health disparities, implicit bias, diversity, culture, social justice, and many other important topics that are frequently lumped together as everything but the basic sciences or much to my chagrin sometimes labeled the “soft skills” of medical education. Encompassing all of these though has been the concept of professionalism.

Perhaps it should not be surprising that my chosen career path of bioethics would lead me to further engagement in professionalism. Medical ethics and humanities teaching have become essential to teaching professionalism in medicine because the concept of professionalism is intrinsically scientific, clinical, ethical, and social ( Doukas at al 2012). Given my role in my medical school’s curriculum, I was offered the chance to serve as a Co-Principal Investigator on an educational study supported by Institute of Medicine as a Profession and the Josiah Macy Jr. Foundation to study social media usage and medical professionalism. Just as I viewed my introduction to bioethics to be serendipitous, so too was this invitation to join senior faculty at my institution to collaborate on the grant. It occurred to me early on in our work that in my own academic training I had never questioned the importance of “professionalism.” I also had not truly thought about *what* it meant to be professional nor was I ever taught *how* to be a professional. All of my former professors, mentors and more senior colleagues assumed that I, then as a student, understood what it meant and expected me to be as such. Now as a medical educator, I realized that few students and faculty could clearly convey their thoughts on the topic either. Professionalism was an accepted, but not thoroughly explored, concept. Our grant examined the challenge for physician educators, who are charged with teaching students about professionalism, which now includes online behavior, but who have little to no familiarity with social media usage ( Kitsis et al 2016).

What it did not do and where I have come to be most concerned today is how do we create a culture that minimizes professional lapses and when they occur addresses them in a meaningful rehabilitative manner. I came to share a concern that medical educators too often transmit to students and residents an unreflective, mechanical and binary view of professionalism. One possible flaw in this teaching is that the concept and practice of professionalism may not be sufficiently grounded in either bioethics or humanities. Such teaching can be a reduction of behaviors into a list of “Do Not’s” based on superficial appearance and comportment, evaluated by checklists ( Brody et al 2014). By focusing only on behaviors and not the context in which those behaviors manifest, we miss an opportunity for remediation and professional growth.

I have come to view professionalism in health care as the representation of behaviors and values that guide us in the care and support of patients, their families and our collaboration with colleagues. Lapses in behavior are rarely intentional, but the result of disinhibition due to lack of situational awareness and coping strategies. Similar to medical error, lapses in professionalism are common, inevitable and occur most often when good people are faced with a challenge that they just don’t have the knowledge, judgment, or skills to address at the time ( Lucey et al 2010). In my experience the reframing of professionalism in this context aligns well with the concept and utilization of Just Culture framework that ensures balanced accountability for both individuals and the organization responsible for designing and improving systems in the workplace ( Boysen 2013). Applying a just culture perspective to professionalism holds individuals accountable for their actions and choices, but also accountable to others, which may help some overcome the inherent resistance to dealing with lapses in professionalism due to impaired or colleagues who have not yet acquired competency ( Wachter 2013). These perspectives have aligned with my own style of coaching in that I don’t consider myself to be an expert on professionalism, but someone committed to facilitating others emerging professional identity and giving them the tools to further develop so that they may continue to grow and learn.

My evolving views and perspectives on medical education and in particular professionalism have not developed in isolation. Thanks to numerous interprofessional collaborations in the course of my academic training and professional work, I have been able to incorporate new ideas and practices into my teaching and scholarly interests. I am incredibly indebted to my colleagues for their collegial generosity. Unbeknownst to them, they were excellent coaches to me and allowed me to maximize my potential. This includes my involvement with the Academy for Professionalism in Health Care (APHC), of which I am a member and currently serve on the Board of Directors. The organization, founded in 2012, was established in response to the Project to Rebalance and Integrate Medical Education (PRIME). It started as a national working group that focused on medical ethics and humanities education as it relates to professionalism education in medical school and residency training in the United States ( Doukas et al 2013).

The theme of this issue asks authors to reflect on and share how they ended up in their career. I mentioned the role of serendipity previously, which I still feel was quite significant for me personally. More important was the willingness to respond to a calling to learning. If not for that, I might not have been exposed to the field of bioethics and later in life the importance of professionalism in teaching. I look forward to where my journey will take me in the future as I embrace the uncertainty of how new and exciting discoveries will alter our societal views of medicine, bioethics and professionalism.

## Take Home Messages

Non traditional career pathway in medical education.

## Notes On Contributors

Patrick Herron, DBe, is a bioethicist and medical educator at Albert Einstein College of Medicine in the Bronx, NY

## References

[ref1] Boysen PG. (2013). Just Culture: A Foundation for Balanced Accountability and Patient Safety. Ochsner J. 2013 Fall;13(3):400–406. PMCID: 24052772 PMC3776518

[ref2] Brody H Doukas D. (2014). Professionalism: a framework to guide medical education. Medical Education 2014. 48:980–987. 10.1111/medu.12520 25200018

[ref3] Carrese JA Malek J Watson K (2015). The Essential Role of Medical Ethics Education in Achieving Professionalism: The Romanell Report Academic Medicine. June 2015-Volume 90-Issue 6- p744–752. 10.1097/ACM.0000000000000715 25881647

[ref4] Doukas D McCullough LB Wear S. (2012). Perspective: Medical Education in Medical Ethics and Humanities as the Foundation for Developing Medical Professionalism. Academic Medicine. March 2012 - Volume 87 - Issue 3- p334–341. 10.1097/ACM.0b013e318244728c 22373629

[ref5] Doukas DJ McCullough LB Wear S (2013). Project to Rebalance and Integrate Medical Education (PRIME) Investigators. The challenge of promoting professionalism through medical ethics and humanities education. Acad Med. 2013;88:1624–1629 10.1097/ACM.0b013e3182a7f8e3 24072126

[ref6] Kitsis EA Milan FB Cohen HW (2016). Who’s misbehaving? Perceptions of unprofessional social media use by medical students and faculty. BMC Medical Education. (2016) 16:67. 10.1186/s12909-016-0572-x 26887561 PMC4757980

[ref7] Lucey C Souba W. (2010). Perspective: The Problem With the Problem of Professionalism. Academic Medicine. June 2010-Volume 85-Issue 6- p1018–1024. 10.1097/ACM.0b013e3181dbe51f 20505405

[ref8] Wachter R. (2013). Personal Accountability in Healthcare: Searching for the Right Balance. The Health Foundation. BMJ Qual Saf. 2013 Feb;22(2):176–180. 10.1136/bmjqs-2012-001227 22942398

