# Supplementary material for: A Calling to Professionalism
Source: MedEdPublish (2016). 2017 Dec 6;6:218. [Version 1] doi: 10.15694/mep.2017.000218 (PMC10885229; doi:10.15694/mep.2017.000218)
Supplement: Peer review history

# Reviewer report: Reviewer response for version 1

**DOI:** 10.21956/mep.19404.r28466

**Published:** 2018-02-02

**Authors:** Susan Van Schalkwyk

**Affiliations:** 1 Stellenbosch University

This review has been migrated. The reviewer awarded 5 stars out of 5 An excellent piece - personal, yet scholarly, well-conceptualised and thoughtful. Much of what the author has written resonated with my own thinking and experience. I suspect that serendipity has featured in the lives of many of us involved in health professions education. I was reminded of how teaching starts with students’ questions, and the importance of exposing students to complexity rather than to offer mechanical, linear solutions. The discomfort expressed around the reference to ‘soft skills’ is shared and conceptualising professionalism as having a scientific, clinical, ethical and social dimension is useful. I look forward to reading more submissions under this theme.

Reviewer Expertise:

NA

No decision status is available

# Reviewer report: Reviewer response for version 1

**DOI:** 10.21956/mep.19404.r28467

**Published:** 2017-12-11

**Authors:** Tan Nguyen

**Affiliations:** 1 Deakin University

This review has been migrated. The reviewer awarded 5 stars out of 5 This article strengthens the importance to keep professionalism at the forefront. I believe, with many other colleagues, that professionalism is probably the most important skillset for all health practitioners to develop and maintain. It is fundamental in all aspects of health care service delivery. What is very difficult in medical education, is how to advance this area of work., both in terms of how to teach it, but also how it is defined, and how we evaluate it.

Reviewer Expertise:

NA

No decision status is available

# Reviewer report: Reviewer response for version 1

**DOI:** 10.21956/mep.19404.r28468

**Published:** 2017-12-08

**Authors:** Gerard Flaherty

**Affiliations:** 1 National University of Ireland Galway

This review has been migrated. The reviewer awarded 4 stars out of 5 I enjoyed this honest reflection from a medical educator who describes his career journey as non traditional. This article raised some interesting points for me. Without a clearly defined career pathway in medical education in many jurisdictions, it takes creativity and perseverance to forge a path. Those who find a way are likely to be dedicated to the art and craft of teaching and they make, as the author describes it, particularly good 'coaches' for medical students, since they themselves have had to apply all their 'softer' qualities to establishing themselves as medical teachers. I wonder if non-traditional should be the norm in this field; I doubt a highly structured postgraduate training scheme in medical education would yield the agile-minded, versatile, supportive educator we wish to provide for our students. The open-minded and flexible approach is very much in evidence in this article. The author challenges some of the traditional and more rigid views on professionalism teaching and adopts a pragmatic philosophy which recognises lapses in professionalism in the context in which they occur. Medical students can view professionalism as a strict set of rules handed down from faceless regulators but this concept may not ultimately serve their life-long development towards becoming honest, reflective, adaptive, resilient, and compassionate professionals. I have one bioethicist colleague and I enjoy my meetings with her over those with all other colleagues, as she teaches me to broaden my frame of understanding of what professionalism teaching should involve. So I salute the non-traditional medical educators, especially those who devote their careers to helping guide medical students towards becoming professionals who are patient-centred and responsive to societal needs.

Reviewer Expertise:

NA

No decision status is available

# Reviewer report: Reviewer response for version 1

**DOI:** 10.21956/mep.19404.r28464

**Published:** 2017-12-07

**Authors:** P Ravi Shankar

**Affiliations:** 1 American International Medical University

This review has been migrated. The reviewer awarded 4 stars out of 5 I enjoyed reading this article on professionalism and the author’s journey as a bioethicist. Professionalism is becoming increasingly important in today’s medical professionalism. In the Caribbean the regional accrediting agency specifically enquires about professionalism and lapses in professionalism. I agree with the author that professionalism may be difficult to define. My former chief academic officer used to mention that while he may not be able to define professionalism he had little difficulty in identifying lapses professionalism. Many of the concepts and ideas behind professionalism may be universal but cultural and social issues could have an influence in many areas of professional behavior. With faster travel and the World Wide Web the world is becoming more closely knit than ever. However, teaching in a Caribbean medical school with a large student body from outside North America I am increasingly reminded of the influence of culture and social values on professionalism. This is an interesting and well-written article which will be of interest to health educators in a variety of settings as professionalism is an overarching concept in healthcare.

Reviewer Expertise:

NA

No decision status is available

# Reviewer report: Reviewer response for version 1

**DOI:** 10.21956/mep.19404.r28465

**Published:** 2017-12-06

**Authors:** Neel Sharma

**Affiliations:** 1 Albert Einstein College of Medicine, Montefiore Medical Center

This review has been migrated. The reviewer awarded 5 stars out of 5 I fully enjoyed reading this piece - and certainly admit to not knowing the field of bioethics in depth. Professionalism as a topic is hugely relevant. Traditionally knowledge and skills were viewed as the fundamentals but now (in the UK at least), professionalism in terms of behaviour/ communication is as important. The biggest source of complaints against doctors in the UK at least is communication based. Furthermore regulators are battling also with a tech savvy workforce and have issued concerns regarding inappropriate social media use for example and how this can be perceived among patients. The junior doctor strike in the UK was viewed by many as unprofessional in a broad sense - discussed particularly during my sabbaticals in Asia where colleagues there culturally have a very strong work ethic regardless. Professionalism will feature more in the future and how best to assess this as well - situational judgement testing being one format - keep up the good work!!!

Reviewer Expertise:

NA

No decision status is available
